# Supplementary material for: Mutations in PCYT2 disrupt etherlipid biosynthesis and cause a complex hereditary spastic paraplegia
Source: Brain. 2019 Oct 22;142(11):3382–97. doi: 10.1093/brain/awz291 (PMC6821184; doi:10.1093/brain/awz291)
Supplement: awz291_Supplementary_Data [file awz291_supplementary_data.zip › awz291-Suppl_data/Supplementary_Data4.zip!/Supplementary information 4 - plasma lipidomics.zip!/Plasma lipidomics/Control vs Patient/Statistical plots and data/LPC species/Volcano plot_alternative.pdf]

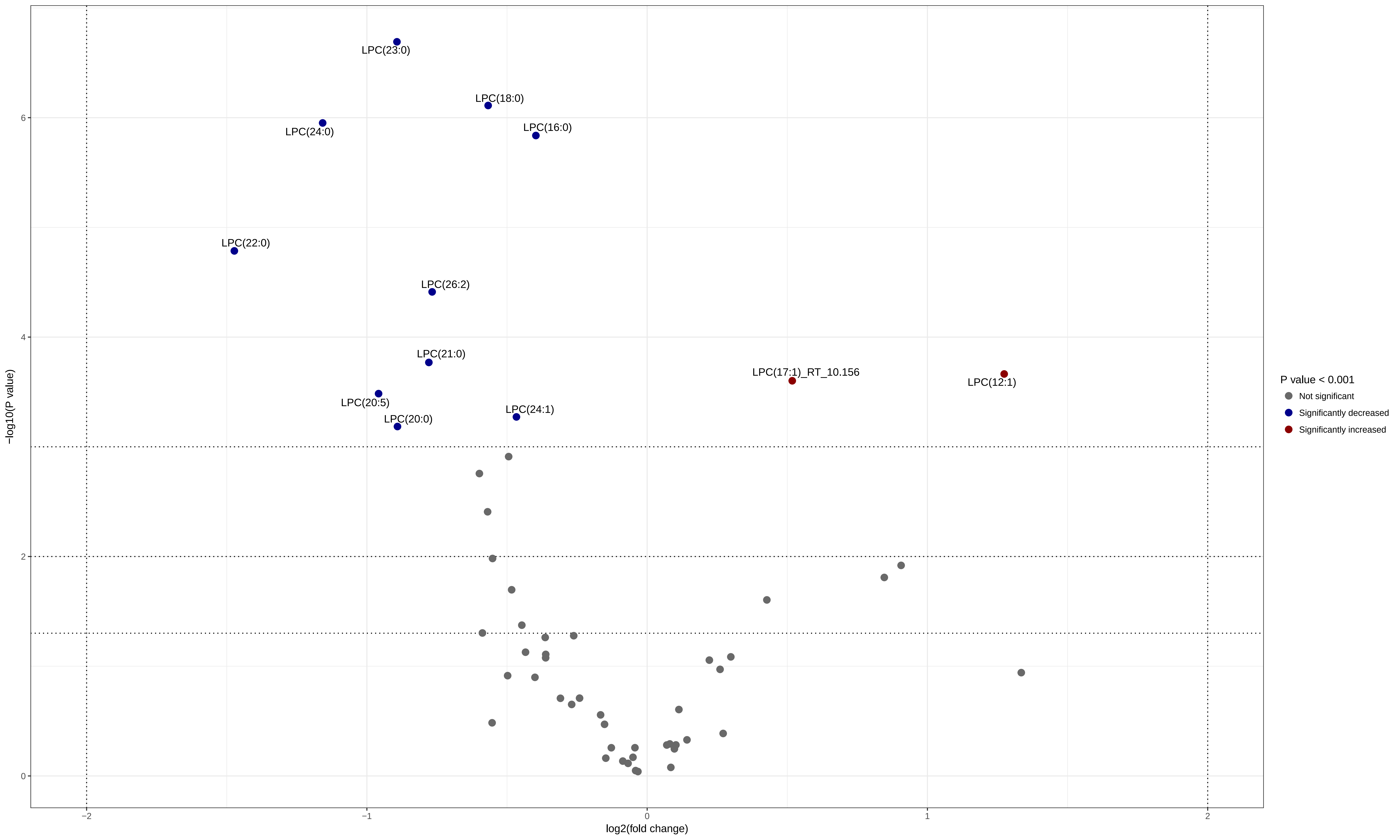
LPC(23:0)
LPC(18:0)
LPC(24:0)
LPC(16:0)
LPC(22:0)
LPC(26:2)
LPC(21:0)
LPC(17:1)_RT_10.156
LPC(12:1)
LPC(20:5)
LPC(24:1)
LPC(20:0)
−log10(P value)
log2(fold change)
P value < 0.001
Not significant
Significantly decreased
Significantly increased
6
4
2
0
−2
−1
0
1
2
